# Comparison of short controlled ankle motion boots and barefoot walking on spatiotemporal gait parameters and plantar pressure distribution

**DOI:** 10.1371/journal.pone.0319671

**Published:** 2025-03-10

**Authors:** Selim Muğrabi, Özlem Feyzioğlu

**Affiliations:** 1 Fulya Foot Surgery Center, Istanbul, Turkey; 2 Department of Physiotherapy and Rehabilitation, Faculty of Health Sciences, Acibadem Mehmet Ali Aydinlar University, Istanbul, Turkey; Brunel University London, UNITED KINGDOM OF GREAT BRITAIN AND NORTHERN IRELAND

## Abstract

Controlled ankle motion (CAM) boots are commonly recommended to protect the foot-ankle complex in reducing loading, continuing ambulation, and maintaining daily activities. However, maintaining a normal and comfortable gait while wearing CAM boots is quite challenging. The added weight of the CAM boot, coupled with reduced ankle work capacity, hinders the full execution of gait parameters, leading to spatiotemporal asymmetry. Different loads on the sole also increase the total mechanical work in the foot. The primary aim of this study was to investigate the impact of short CAM boots on spatiotemporal gait parameters and plantar pressure distribution. Twenty-four healthy participants were recruited for the study. The participants were asked to walk barefoot and wear bilateral short CAM boots at their comfortable speed. Spatiotemporal gait parameters, foot-pressure distribution, and force were evaluated with Zebris FDM-THM-S treadmill system (Zebris Medical GmbH, Germany) under both conditions, the right and left extremities were evaluated independently. One-way Analysis of Variance (ANOVA) was used to compare the spatiotemporal characteristics of the participants. Significant differences were observed between barefoot and CAM boot walking for all parameters(p < 0.05), except walking speed (p > 0.05). Short CAM boots walking showed a notable increase in the forefoot, midfoot, and hindfoot pressure distribution, with the highest rise in the midfoot region (p < 0.05). Short CAM boots cause an increase in pressure of the entire sole, therefore, caution should be taken before recommending this device, particularly in midfoot pathologies.

## 1. Introduction

Controlled ankle motion (CAM) boots are often prescribed for post-surgical immobilization following traumatic injuries of the foot and ankle, such as Achilles tendon rupture, and for wound load relief in conditions such as diabetic foot ulceration [1,2]. CAM boots allow patients to enable ambulation and other activities of daily living while unloading and protecting the ankle and foot complex [2,3]. They provide more functional benefits than plaster casts, and are also removable, making them more hygienic. Despite their functional benefits, maintaining normal and comfortable walking in CAM boots poses challenges as they restrict joint movements to provide stability and distribute weight effectively [4]. CAM boots are designed to restrict ankle and foot movements and provide a stable platform to distribute forces when weight-bearing [5–7]. Most controlled walking boots are designed to grip the extremity and allow the patient to rotate the foot and ankle complex while walking. Thus, walking boots protect the ankle and foot, allowing the return to ambulation with continuous protection of the injured extremity [2,8].

Various pathologies are frequently managed using different types of CAM boots. “Tall” CAM boots are generally preferred for conditions requiring enhanced immobilization, where the foot is immobilized during the initial two weeks [1,9]. In contrast, “short” CAM boots are often prescribed due to their increased convenience and are particularly suitable for conditions that primarily require offloading [10].Although CAM boots are commonly used in the rehabilitation of orthopedic diseases, they can cause secondary problems [8]. Studies have shown gait biomechanics change with the restriction of ankle and foot function. Additionally, patients may experience difficulty in maintaining postural stability and suppressing exaggerated body movements reflected by assessment of balance and range of motion (ROM) [11,12]. Nahm et al. reported that both short and tall CAM boots effectively reduce ROM; however, the short CAM boot is comparatively less restrictive [13]. This decreased restriction may, reduce the risk of secondary joint pain and muscle atrophy in the lower extremity muscles [14], potentially minimizing long-term structural and architectural deficits in the musculature of the foot and ankle complex [15]. Furthermore, tall CAM boots increase the mass of the lower limb, causing it more difficult to lift and leading to greater fatigue in the muscles responsible for limb propulsion, particularly during the early and mid-swing phases of gait [16]. Therefore, the determination of spatiotemporal gait parameters of short CAM boots could be beneficial for clinicians.

Studies comparing the pressure distributions of various foot and footwear equipment have indicated that controlled walking boots reduce the mean plantar peak pressure and contact pressure during the stance phase of walking [17]. Previous research has compared, the CAM boots with different insoles or running shoes showing to significantly reduced plantar pressure compared to standard athletic footwear [18]. Although it has been reported that controlled gait boots provide mechanical advantages in gait parameters and sole pressure distributions, the difference they may cause in the normal walking period remains unknown [5].Notably, short CAM boots have been shown to be more effective than tall CAM boots in maintaining a more natural gait, but the amount of deviation from normal gait remains unclear [15]. This study aims to investigate the differences in step length, stride length, step width, gait velocity, stance and swing phase percentages, cadence parameters, and sole pressure distribution in healthy individuals wearing CAM boots, which are commonly prescribed following foot surgeries. We hypothesize is that the short CAM boots alter spatiotemporal gait parameters and plantar pressure distribution in unexpected way on the forefoot, midfoot and hindfoot.

## 2. Materials and methods

### 2.1. Study design and subjects

The present study was designed as a cross-over study. A total of 24 participants, who applied to Fulya Foot Surgery Clinic between January and May 2024 were recruited. Ethical approval was obtained from the Acibadem University ethics committee (ATADEK 2023-16/564), and the study adhered to the principles of the Declaration of Helsinki.

Healthy participants aged 20-40 years who did not have any musculoskeletal injuries or neurological conditions that could affect gait we included in the study during the data collection. Participants were excluded if they had peripheral neuropathy, neurological or orthopedic pathology affecting balance coordination, preexisting musculoskeletal impairments, history of lower extremity surgery and, history of foot trauma. All participants provided written informed consent prior to the study.

### 2.2. Procedure

**Fig 1:**
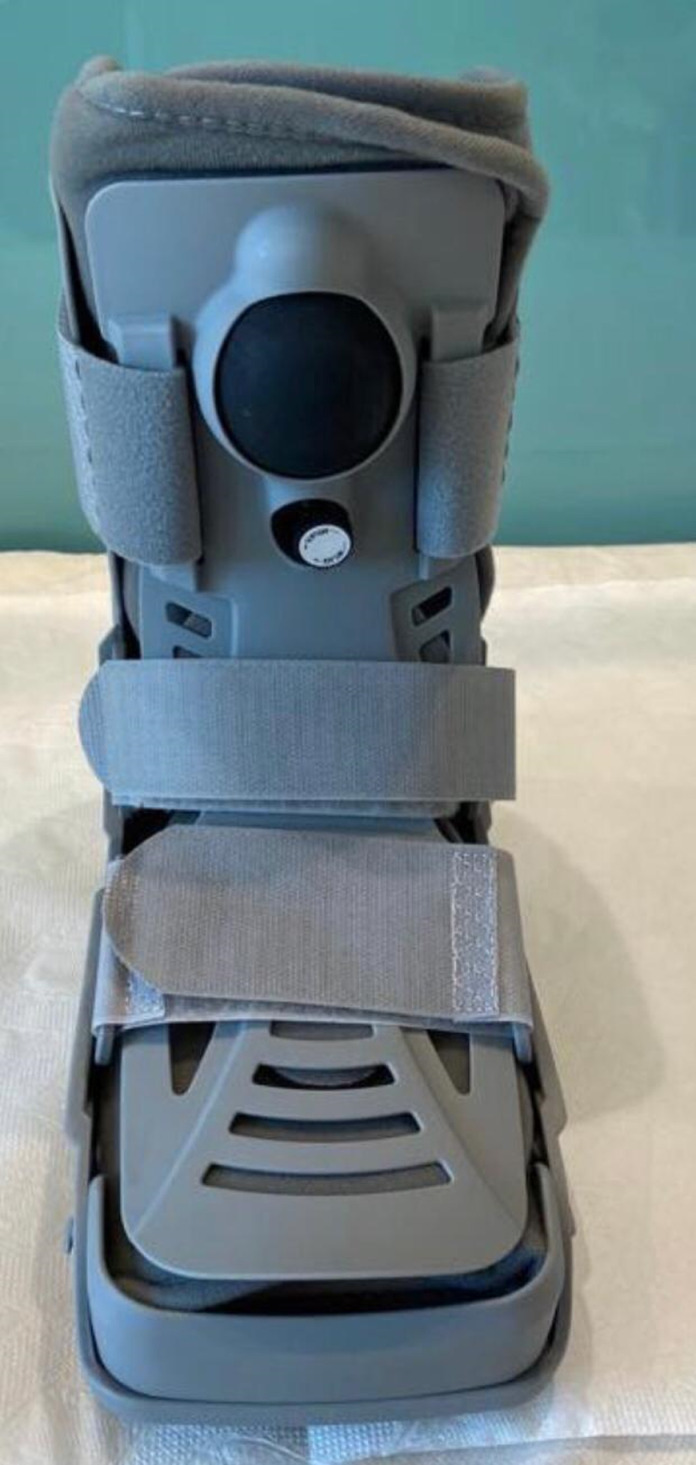
Short CAM Boot.

Participants were instructed to walk barefoot at a comfortable speed on a treadmill in a horizontal position. Spatiotemporal gait parameters and foot pressure distribution of the participants were evaluated with the Zebris FDM - THM - S (Zebris Medical GmbH) treadmill system. After a 10-minute walking period, participants rested for 2 hours for the washout period. Subsequently, CAM walking boots (protect.CAT®Walker, Deutschland) were put on, and the test was repeated. CAM walking boot has a flat, rounded, and slip resistant sole, an integrated air pump for pressure regulation and it can support normal gait. The protect CAT Walker boot has the capability to limit the anterior displacement of the talus with a strap. An integrated air pump and the air release valve ensure that the user is able to regulate the pressure easily (Fig 1).

Participants were informed that the test could be terminated if they felt discomfort in the boots or pain due to the weight of the boots. The measurement duration was set to 10 minutes, with the first 6 minutes unrecorded. During the subsequent 4-minute walking period, foot pressure distribution and gait parameters were recorded for 10 seconds. On average, the mean of 14–18 steps were calculated for each participant. To avoid the learning effect of walking with CAM boots, practicing before the test was not allowed. All assessments were performed by an assessor blinded to the study.

### 2.3. Measurement

#### 2.3.1. Outcome measurement.

**2.3.1.1. Spatial-temporal gait parameters and foot pressure distribution:** Changes in gait parameters and foot pressure distribution were evaluated using the Zebris FDM – THM – S (Zebris Medical GmbH) treadmill system. The Zebris FDM-THM-S system includes 0.85 cm *  0.85 cm force sensors placed on the floor of the treadmill that can not be seen from the outside. The treadmill has a contact surface of 150 cm by 50 cm and an adjustable speed range from 0.2 km/h to 22 km/h [19]. When the participants stand or walk on the treadmill, the sensors can measure reactive and normal force in three dimensions at a frequency of 120 Hz. Due to the high density of the sensors, even the smallest changes in the pressure distribution are analyzed and pressure maps are produced. Timing can be monitored during the process. By integrating the signals from the force sensors, the system provides comprehensive temporal and spatial gait parameters, including pressure center trajectories during static standing and walking. These parameters are provided in two or three dimensions [20]. The Zebris FDM-THM-S treadmill system has demonstrated validity and high reliability in analyzing spatial and temporal gait parameters and pressure distribution evaluations [19,21].

The participants were given the first 6 minutes as practice time on the treadmill. During the next 4 minutes of the walking period, foot pressure distribution values and gait parameters were recorded for 10 seconds.

### 2.4. Sample size and statistical analysis

The sample size of the study was determined based on the baseline pressure value in the study conducted by Hunt et al [5] with alpha 0.05 for a power of 0.95, it was calculated that a total of 24 participants were required. The sample size was calculated using the GPower V.3.1.7 program (Kiel University, Kiel, Germany). The normality of the data was analyzed using the Shapiro-Wilk test. Descriptive statistics for quantitative variables were presented as median and interquartile range or mean and standard deviation, while frequency and percentage were used for qualitative variables. One-way Analysis of Variance (ANOVA) was used to compare the spatiotemporal characteristics of the participants between right barefoot, left barefoot and right CAM boot and left CAM boot groups with post-hoc Bonferroni corrections. In addition The Games–Howell method is applicable in cases where the equivalence of variance assumption is violated. P value at < 0.05 was considered as significant. All statistical analyses were conducted using SPSS 21.0 for Windows (IBM Inc., Armonk, NY, USA).

## 3. Results

Our study was conducted with 24 healthy participants. All participants completed the two tests without any problems. The demographic characteristics of the participants are presented in Table 1. Of 24 participants 32.35% were women and, all participants were right- handed. ANOVA analysis revealed no significant difference between right and left barefoot walking or between right and left CAM boot walking (p > 0.05). However, a significant difference was observed between walking with the CAM boot and walking barefoot (p < 0.001), regardless of the right and left side, in all parameters except walking speed. CAM boot walking showed a significant increase in step length, stride length, and stride width. Additionally, walking with the CAM boot led to a decrease in the stance phase, loading response, and pre-swing phase and a significant increase in the swing phase, while it decreased cadence (p < 0.001) without affecting gait speed (Table 2).

**Table 1:** Demographic variables of patients.

Variables	Barefoot-CAM boot (n = 24)
	Mean ± SD
Age (years)	35.04** ±** 9.34
Height (cm)	168.94** ±** 7.05
Body weight (kg)	73.42** ±** 12.18
BMI (Kg/m^2^)	25.60** ±** 3.06
Female/ male	9/15

CAM, Controlled ankle motion; BMI, Body Mass Index; SD, Standard Deviation.

p* values obtained from the Independent Samples t test.

**Table 2:** Spatio-Temporal Gait Parameters and Foot Pressure Distribution in Different Conditions.

Variables	Barefoot Left (n = 24) Mean ± SD	Barefoot Right (n = 24) Mean ± SD	CAM Boot Left (n = 24) Mean ± SD	CAM Boot Right (n = 24) Mean ± SD	*F*	P*
***Gait parameters***						
Step length (cm)	45.02 ± 9.48	46.62 ± 4.35	51.54 ± 6.12	51.79 ± 4.70	6.731	**.000**
Stride length (cm)	93.66 ± 8.38		102 ± 11.34		5.58	**.001**
Step width (cm)	10.33 ± 3.14		15 ± 2.68		20.37	**.000**
Stance phase percentage (%)	65.76 ± 1.20	66.10 ± 1.90	61.50 ± 4.77	62.16 ± 5.35	9.66	**.000**
Loading response (%)	15.75 ± 1.75	16.15 ± 1.34	11.74 ± 4.78	11.97 ± 4.20	11.89	**.000**
Mid stance (%)	33.89 ± 1.90	34.15 ± 1.20	37.85 ± 4.83	38.03 ± 4.96	9.28	**.000**
Pre swing (%)	16.16 ± 1.35	15.75 ± 1.73	11.85 ± 4.63	11.89 ± 4.55	11.42	**.000**
Swing phase percentage (%)	34.20 ± 1.14	33.90 ± 1.90	38.49 ± 4.77	37.83 ± 5.35	9.77	**.000**
Step time, sec	0.61 ± 0.06	0.62 ± 0.62	0.69 ± 0.64	0.68 ± 0.65	7.54	**.000**
Stride time, sec	1.24 ± 0.12		1.35 ± 0.14		4.80	**.004**
Gait speed (m/s)	2.70 ± 0.10		2.71 ± 0.06		0.16	.919
Cadance (steps/min)	97.33 ± 10.14		88.04 ± 8.08		8.21	**.000**

**Fig 2:**
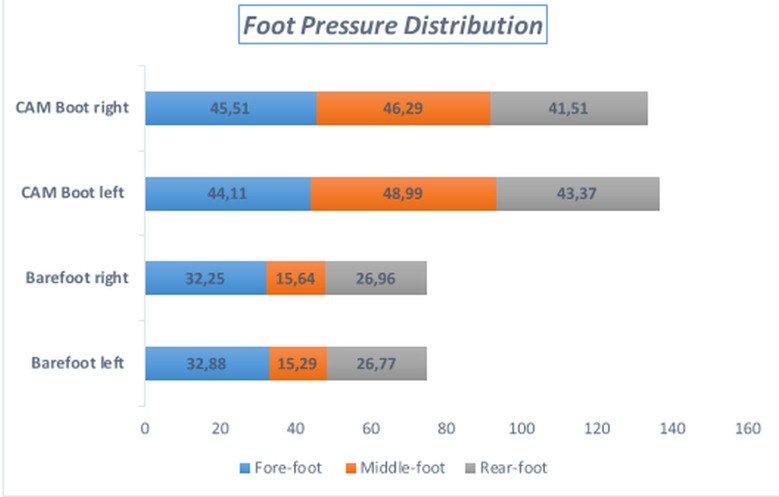
Foot Pressure Distribution.

CAM boot increased plantar pressure distribution on all areas of the foot significantly (p < 0.001) and the highest mean differences were shown in the middle foot regardless of side (Fig 2). When comparing the forces in the forefoot, midfoot, and rearfoot, the CAM boot increased force in the midfoot while decreasing it in the rearfoot (p < 0.05) (Table 3).

**Table 3:** Spatio-Temporal Gait Parameters and Foot Pressure Distribution in Different Conditions.

Variables	Barefoot Left (n = 24) Mean ± SD	Barefoot Right (n = 24) Mean ± SD	CAM Boot Left (n = 24) Mean ± SD	CAM Boot Right (n = 24) Mean ± SD	*F*	P*
**Foot pressure distribution** ***(N/cm2)***						
Forefoot	32.88 ± 6.50	32.25 ± 6.67	44.11 ± 11.08	45.51 ± 12.45	13.25	**.000**
Middlefoot	15.29 ± 4.71	15.64 ± 4.51	48.99 ± 12.22	46.293 ± 12.24	97.24	**.000**
Rearfoot	26.77 ± 5.11	26.96 ± 5.17	43.37 ± 16.19	41.56 ± 12.58	16.55	**.000**
***Foot force (N)***						
Forefoot	649.81 ± 167.93	653.17 ± 139.00	576.64 ± 154.57	576.02 ± 168.74	1.81	.151
Middlefoot	156.69 ± 66.72	168.38 ± 71.66	642.45 ± 123.55	632.64 ± 152.17	150.46	**.000**
Rearfoot	465.27 ± 92.06	464.73 ± 81.32	396.37 ± 166.98	387.00 ± 118.38	3.04	**.033**

## 4. Discussion

The objective of this study was to investigate the effects of the short CAM boot on gait parameters, foot pressure, and foot force in healthy participants. Our findings indicate that CAM boots changed all spatiotemporal gait parameters except for the gait speed. In addition, CAM boot increased plantar pressure in the forefoot, middle foot, and rear foot while reducing force in the rearfoot.

Wearing CAM boots affects the swing phase, potentially extending its duration [10]. Unilateral CAM boot gait prolongs the duration of the single support phase, particularly on the ipsilateral side. Consequently, an extended midstance phase is anticipated. In the present study, the loading response and pre-swing phases were reduced, while the midstance phase was increased during walking with the boot [22].

Sommer et al. conducted a comparison of three different ankle stabilization boots and observed a significant reduction in step length on the booted side compared to the contralateral side. Keene et al reported that walking boots cause postural and lower limb instability, leading patients to walk with widened steps to provide additional gait stability [23]. In our study, walking with a CAM boot provided a significant increase in step length, stride length, and width. However, while previous studies typically involved unilateral CAM boots, our participants used bilateral boots, which, yielded similar results. Furthermore, the boots used in the previous studies exhibited different properties. This heterogeneity can lead to limitations and potential inconsistencies in interpreting the results.

Reduced ankle ROM in CAM boots also diminishes or eliminates the capacity of the ankle to produce effective joint moments. However, these moments have been shown to contribute significantly to the overall biomechanical work during walking at various speeds [24, 25]. Keene et al. found that walking speed in CAM boots was higher than in the Tubigrip and they also found similar results in the comparison of stirrup and CAM boot [23]. In previous studies evaluating the efficacy of boots, no fixed walking speed was prescribed. Instead, participants were instructed to walk at a self-selected speed. These studies reported a significant reduction in walking speed when using boots compared to controls. The analyses revealed that the average walking speed with boots was 1.34 m/s in women and 1.43 m/s in men. In our study, there was no significant difference between barefoot and CAM boot walking in terms of gait speed. In addition, walking speed was higher than values observed in previous literature for two conditions. We believe that this result is due to the use of the short CAM boot. The consistent walking speed observed in both conditions may result in more reliable findings regarding the other spatiotemporal parameters evaluated.

Contrary to the results of the current study, Chen et al found no significant differences in spatiotemporal gait variables of stroke patients under different conditions as barefoot and ankle foot orthosis [26]. However, it was observed that the cadence was the highest during barefoot walking aligning with studies comparing barefoot walking with various footwear, where walking boots reduced cadence [27]. Previous research has compared, barefoot walking and walking with different footwear and orthotic approaches, showing contradictory results in terms of cadence. However, cadance was found to be highest in barefoot walking. The decrease in the number of steps without changing the speed may be explained by the increase in the step length in walking with a CAM boot. The mechanical weight of CAM boots may also be effective in decreasing cadence. The effect of increased mechanical weight causes an excessive workload on the muscles responsible for lifting the limb during the early and mid -swing phase. This is alternative explanation of the reduction in the number of steps [22].

The functions of walking boots include immobilization, protecting the ROM, reducing the load on specific foot regions, minimizing pain and allowing safe treatment with and without surgery. In an investigation of various foot orthoses, it was found that the use of a long walking boot significantly reduced plantar pressure in the forefoot region without affecting the pressure distribution in the midfoot. In contrast, the short CAM boot was observed to decrease forefoot pressure while increasing pressure in the midfoot. [28]. Findings from CAM boot studies focused on the reduction of pressure in the forefoot but conflicting evidence suggests that it moderately reduces or increases heel pressure [29,30]. Our findings showed that short CAM boots increased pressure in all areas of the foot. According to these results, increased pressure in all regions of the sole raises questions about the suitability of the CAM boots and, its potential applicability in different pathologies.

Traditional CAM boots have shown similar results in terms of force offloading capabilities compared to athletic shoes. It has been reported that the spring CAM boot is effective in absorbing ground reaction force and force transmission [10]. However, it was observed that force offloading was higher in CAM boot walking compared with barefoot walking. Especially in the forefoot and hindfoot ulcers, the traditional CAM boot can transfer force more effectively than barefoot [31]. Our results showed a significant reduction in forefoot and rearfoot force, consistent with the literature, but a dramatic increase in midfoot force. The collection of all forces in the midfoot reveals that CAM boot should be used or selected carefully, especially in midfoot pathologies. The fact that CAM boot provides similar results in terms of force distribution with an athletic shoe may make it more advantageous to select athletic shoes over heavy mechanical CAM boots in foot pathologies without ankle problems. Additionally, spring-loaded walker boots are reported to be more effective in force transfer, presenting another alternative to traditional CAM boots [10].

Walking boots also limit sagittal ankle motion, decreasing muscle activity and potentially leading to muscle atrophy in the gastrocnemius, soleus, and peroneal muscles. Muscle atrophy contributes to a prolonged rehabilitation process, which delays the return to training in various pathologies [32,33]. Therefore, clinicians need to decide the efficacy of traditional CAM boot based on a patient and identify situations to maximize benefit.

The present study has some limitations. First, it was conducted only in healthy individuals, and differences between bilateral and unilateral boot use in individuals with ankle or foot pathology were not investigated. Second, the participants only walked at the speed they felt comfortable and the effects of walking at different speeds were not examined. Third, we could not do randomization, the first 24 volunteer participants who applied to the clinic were included in the study. Finally, we investigated the acute effect of CAM boot because participants were allowed 10 minutes to become acclimated to the CAM boot condition. Using long-term boots could affect different gait parameters, so randomized controlled trials over multiple weeks are needed to understand the long-term effects of CAM boots on gait parameters.

## 5. Conclusion

The findings from this study indicated that traditional short CAM boots increased step length, step wide, and swing phase percentage while decreasing stance phase percentage and cadence. In addition, the short CAM boot increased pressure on the forefoot, midfoot, and rearfoot; however, it was only effective in reducing force on the rearfoot. Future research should compare short traditional CAM boots with spring adaptive walking boots and barefoot walking in various foot pathologies, particularly midfoot conditions.
